# Prognostic factors in gastric cancer patients: a 10-year mono-institutional experience

**DOI:** 10.3389/fsurg.2024.1321981

**Published:** 2024-02-01

**Authors:** N. De Ruvo, S. Fenocchi, L. Veronesi, G. Missori, A. A. Ricciardolo, E. G. Rossi, L. Sorrentino, N. Cautero, F. Serra, R. Gelmini

**Affiliations:** General, Oncological and Emergency Surgery Unit, Department of Surgery, AOU Policlinico di Modena, University of Modena and Reggio Emilia, Modena, Italy

**Keywords:** gastric cancer, gastrectomy, prognostic factors, risk factor, survival after surgery

## Abstract

**Introduction:**

Gastric cancer (GC) is one of the main causes of death from cancer globally. Long-term survival, especially in Western countries, remains dismal, with no significant improvements in recent years. Therefore, precise identification of clinical and pathological risk factors is crucial for prognosis, as it allows a better selection of patients suitable for oncologically radical treatments and contributes to longer survivals.

**Methods:**

We devised a retrospective observational longitudinal study over 10 years of experience with GC patients operated with curative intent.

**Results:**

Several factors were thoroughly investigated in a multivariate analysis to look for significance as independent risk factors for disease-free survival. Our results showed that only BMI, pTNM, and lymph node ratio expressed hazard ratios with implications for survival in our series of patients.

**Discussion:**

Although limited by the retrospective nature of the study, this is one of the few cancer reports from Northern Italy showing results over 10 years, which may in our view, have an impact on decision-making processes for multidisciplinary teams dedicated to the care of gastric cancer patients.

## 1 Introduction

Gastric cancer (GC) is one of the main causes of death from cancer globally; in fact, it is the fifth most common neoplasm and the third most lethal worldwide (GLOBOCAN 2018) (1). Its development process is linked to both environmental and genetic factors, with as much as 50% of cases related to dietary habits and social behaviours (2). Moreover, up to 3% of cases may be attributed to inherited cancer-predisposing syndromes (3). Although its incidence has been on the decline in the last few decades, long-term survival remains dismal, especially in Western countries, with no significant improvements in recent years (4).

Currently, the globally accepted gold standard for GC treatment is radical surgery (R0 gastrectomy + D2 lymphadenectomy) (5, 6). In most cases, surgery alone does not represent a radical treatment, so perioperative treatments like neoadjuvant or adjuvant chemo-radioimmunotherapy are recommended for achieving long-term overall survival (OS) (7).

Unfortunately, up to 75% of GC patients are diagnosed when the disease has reached a locally advanced or metastatic stage; therefore, median OS rarely exceeds 12 months in the metastatic setting, and the 5-year OS remains lower than 10%.

Therefore, the correct identification of clinical and pathological risk factors is crucial for prognosis, as it allows a better selection of patients suitable for oncologically radical treatments and contributes to longer survivals.

The objective of our study was to evaluate the impact of clinical, pathological, and treatment-related risk factors on the survival outcomes of patients with GC who underwent operative procedures at a tertiary referral centre in Northern Italy from 2009 to 2019.

## 2 Material and methods

### 2.1 Patient selection

Medical records of all GC patients operated on at the Policlinico of Modena between 2009 and 2019 were collected through a prospective electronic database and reviewed retrospectively. Each patient’s record included demographic information, along with clinical, laboratory, pathological, surgical, and oncological therapy data.

A series of key variables were analysed, including age, sex, Eastern Cooperative Oncology Group (ECOG) performance status (PS), preoperative BMI, Helicobacter pylorii (HP) infection, tumour site, symptoms if relevant, pathological data [type of tumour, T, N, lymph node ratio (LNR), metastasis if any, grading, resection margins, angiovascular and perineural invasion, disease stage, HER2 status], haematological markers of inflammation [white blood cell, neutrophil, lymphocyte, and platelet count, neutrophil–lymphocyte ratio (NLR), platelet–lymphocyte ratio (PLR), and lymphocyte–monocyte ratio (LMR)], carbohydrate antigen 19-9 (CA19-9), and carcinoembryonic antigen (CEA). Surgical data regarding the type of surgery performed and the onset of postoperative complications were also retrieved. Complications, if they occurred, were classified by the Clavien–Dindo classification. From an oncological perspective, data on the administration of chemotherapy (perioperative or adjuvant), outcomes (recurrence or death), and the duration of follow-up in months were also recorded. All patients were staged according to national guidelines (8, 9), including blood examinations, CEA and CA19-9 tumour markers, esophago-gastro-duodeno-scopy (EGDS) plus tumour biopsy, and a whole-body CT scan with contrast medium. Therefore, an institutional GC Multi-Disciplinary Team planned the best therapeutic pathway, which includes up-front surgery with curative or palliative intent, perioperative chemotherapy, or best supportive care.

All selected patients had been scheduled for an elective standard gastrectomy plus D2 lymphadenectomy; patients with urgent operation requirements and a follow-up duration of less than 6 months were excluded from the analysis. Histologies other than adenocarcinoma were also excluded. All pathological reports were revised to establish the TNM staging according to the last TNM classification [UICC classification, 8th ed. (10)].

The study protocol conformed to the ethical guidelines of the 1975 Declaration of Helsinki. Data were collected under protocol 1186-2018/OSS/AOUMO, which was reviewed and approved by the Ethics Committee of the Area Vasta Emilia Nord.

### 2.2 Statistical analysis

In this study, descriptive statistics are reported as proportions or medians with interquartile ranges (IQRs). Differences in the distribution of characteristics between groups were studied using the chi-squared test or Fisher exact test to compare categorical variables. The Mann–Whitney *U*-test was used to compare continuous variables that were not normally distributed, while the Student *t*-test was used in all other cases. The primary outcome was disease-free survival (DFS), which is defined as the time from the day of operation to the date of recurrence or death, whichever is first. The Kaplan–Meier method (KM) was utilised to estimate survival, and the log-rank test was used for assessing significant differences. To determine the existence of prognostic factors, data on DFS were subjected to univariate analysis using the Cox proportional hazard method. Significant variables were selected, and multivariate analysis was finally performed to weigh different hazard ratios, determining their significance as independent prognostic factors for survival.

Statistical significance was determined if the *p*-value was <0.05. The entire statistical analysis was performed with the SPSS statistical program (version 25.0).

## 3 Results

**Figure 1:**
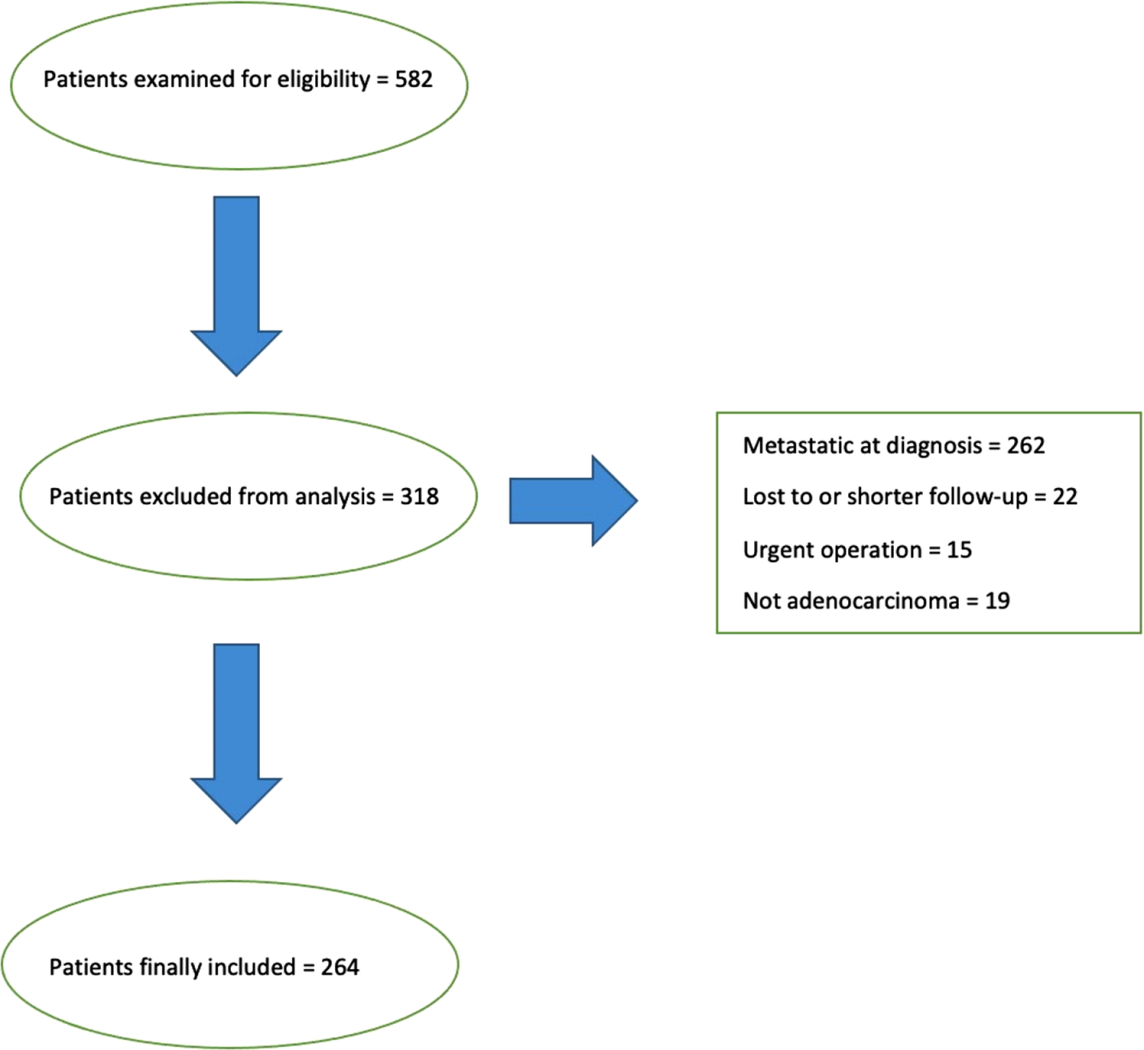
Inclusion and exclusion criteria and final population.

A total of 264 patients, comprising 115 women and 149 men, who underwent surgery for GC at the Policlinico of Modena between 2009 and 2019, were included in the analysis. Other 277 patients with known metastatic disease at the time of diagnosis were excluded (see Figure 1). In a small group of 29 (10.9%) patients, a limited peritoneal involvement of the tumour became apparent only during surgery. These cases were considered in the final analysis (real-life analysis).

### 3.1 Patient characteristics

The median age was 73 years (range 20–94), with an ECOG PS of 0–1 observed in over 80% of patients; the tumour was located at the gastro-esophageal junction (GEJ) in as low as 11.4% of cases, whereas adenocarcinoma of intestinal type was identified in 56.8% of cases; 59.8% received a subtotal gastric resection; and a standard D2 lymphadenectomy was performed in the majority of cases (94.3%), with an average of 27.4 (range 15–86) retrieved lymph nodes for examination. During the operation, 10.9% of patients were accidentally discovered to have limited intra-abdominal peritoneal metastasis; these patients underwent radical surgery anyway (R0) and subsequently received postoperative chemo- or chemoradiotherapy. In 34 cases (12.7%), resection margins were classified as microscopically positive (R1); final histologic examination showed G3 in a predominant 68.2% of all cases and a pTNM stage III in 112 patients (44%). Before surgery, 18.2% of patients received neoadjuvant chemotherapy, whereas after the operation, 50.9% of all patients received adjuvant treatments.

Table 1 shows the basal characteristics of patients grouped by occurrence of recurrence (disease-free or not). Continuous variables for age, NLR, LMR, PLR, CA19-9, and CEA are reported as medians (IQR in parentheses); the remaining are given as numbers (percentage in parentheses).

**Table 1:** Clinical, surgical, and pathological characteristics of patients.

Characteristics	Total (*n* = 264)	REC No (*n* = 161; 61%)	REC Yes (*n* = 103; 39%)	*p*
Age (years)	73 (15)	71 (11)	69 (10)	ns
Sex				ns
Male	149 (56.4%)	91 (61%)	58 (39%)	
Female	115 (43.6%)	69 (60%)	46 (40%)	
BMI (kg/m^2^)	25 (5.4)	24.4 (4.6)	26.1 (3.8)	0.02
Class I (≤18.5)	36 (13.7%)	13 (36.1%)	23 (63.9%)	
Class II (>18,5 < 25)	108 (40.9%)	68 (62.9%)	40 (37.1%)	
Class III (≥25)	120 (45.4%)	56 (46.5%)	64 (53.5%)	
ECOG PS				ns
0	79 (29.9%)	55 (69.6%)	34 (30.4%)	
1	164 (62.1%)	95 (57.9%)	69 (42.1%)	
2	15 (5.7%)	6 (40%)	9 (60%)	
3	6 (2.3%)	4 (66.6%)	2 (33.4%)	
Tumour site				ns
Upper	30 (11.4%)	15 (50%)	15 (50%)	
Lower	225 (85.2%)	140 (62.2%)	85 (37.8%)	
Diffuse laminitis	9 (3.4%)	6 (66.6%)	3 (33.4%)	
NLR	1.99 (1.51)	1.8 (0.9)	2.2 (1.5)	ns
LMR	4.3 (2.69)	4.01 (2.4)	4.2 (2.6)	0.001
PLR	148.3 (75.8)	139.3 (86.4)	164.0 (66.6)	0.001
CA19-9	9.3 (16.0)	7.5 (19.3)	11.1 (17.4)	ns
CEA	1.4 (1.8)	1.35 (1.97)	1.6 (1.6)	ns
Type of resection				ns
Subtotal	158 (59.8%)	100 (63.3%)	58 (36.7%)	
Total	93 (35.3%)	52 (56%)	41 (44%)	
Expl. lap.	13 (4.9%)	9 (6.9%)	4 (3.1%)	
pTNM				0.0001
IA	37 (14)	36 (97.2%)	1 (2.8%)	
IB	25 (9.5)	21 (84%)	4 (16%)	
IIA	37 (14)	27 (73%)	10 (17%)	
IIB	36 (13.7)	21 (58.3%)	15 (41.7%)	
IIIA	50 (18.9)	24 (48%)	26 (52%)	
IIIB	38 (14.4)	17 (44.7%)	21 (55.3%)	
IIIC	41 (15.5)	15 (36.5%)	26 (63.5%)	
Histotype				0.001
Intestinal	150 (56.8%)	101 (67.3%)	49 (32.7%)	
Diffuse	92 (34.8%)	45 (48.9%)	47 (51.1%)	
Mixed	22 (8.4%)	15 (68.1%)	7 (31.9%)	
Grading				0.002
I	10 (3.8%)	8 (80%)	2 (20%)	
II	64 (24.2%)	43 (67.2%)	21 (32.8%)	
III	190 (72%)	100 (52.6%)	90 (47.4%)	
Vascular invasion				0.003
Yes	153 (58%)	79 (51.6%)	74 (48.4%)	
No	111 (42%)	77 (69.3%)	34 (30.7%)	
R positivity				ns
Yes	42 (16%)	21 (50%)	21 (50%)	
No	222 (84%)	135 (60.8%)	87 (39.2%)	
Perineural invasion				0.01
Yes	108 (41%)	53 (49%)	55 (51%)	
No	156 (59%)	103 (66%)	53 (34%)	
LN positivity				0.0001
Yes	179 (67.8%)	85 (47.4%)	94 (52.6%)	
No	85 (32.2%)	74 (87%)	11 (13%)	
LNR (lymph node ratio)				0.001
I (0)	76 (28.7%)	68 (89.4%)	8 (10.6%)	
II (≤0.1)	44 (16.6%)	27 (61.3%)	17 (38.7%)	
III (>0.1 ≤ 0.25)	45 (17.1%)	22 (48.8%)	23 (51.2%)	
IV (>0.25)	99 (37.6%)	40 (40.4%)	59 (59.6%)	
Adjuvant chemotherapy				0.001
Yes	133 (50.3%)	59 (44.3%)	74 (55.7%)	
No	131 (49.7%)	102 (76.7)	29 (23.3%)	
Perioperative chemotherapy				ns
Yes	48 (18.2%)	25 (52%)	23 (48%)	
No	216 (81.9%)	136 (63%)	80 (37%)	
Complications				0.001
0	171 (64.8%)	92 (54%)	79 (46%)	
I	36 (39%)	28 (77.8%)	8 (22.2%)	
II	42 (45.1%)	29 (69%)	13 (31%)	
IIIA	6 (6.4%)	6 (100%)	0 (0%)	
IIIB	6 (6.4%)	4 (66.7%)	2 (33.3%)	
IVA	2 (2.1%)	1 (50%)	1 (50%)	
V	1 (1%)	1 (100%)	0 (0%)	

Continuous variables for age, NLR, LMR, PLR, CA19-9, and CEA are reported as medians (IQRs), and the remaining are given as numbers (percentages). REC, recurrence.

### 3.2 Disease-free survival analyses

The mean (median) follow-up period was 35 (26) months for the overall series. The longest period of follow-up observed from surgery to the time of closure of the study has been 121 months.

The mean (median) DFS was 65.65 (52.0) months [95% confidence interval (CI): 57.9–73.3 (26.0–77.9)], and 3-, 5-, and 7-year DFS scores were 56.1%, 49.6%, and 45.2%, respectively. There was a total of 103 recurrences (39%).

Risk factors significantly associated with DFS were BMI, histotype, LNR, pTNM, grading, vascular budding, perineural and margin infiltration, NLR, PLR, LMR, and finally postoperative chemotherapy.

#### 3.2.1 BMI

**Figure 2:**
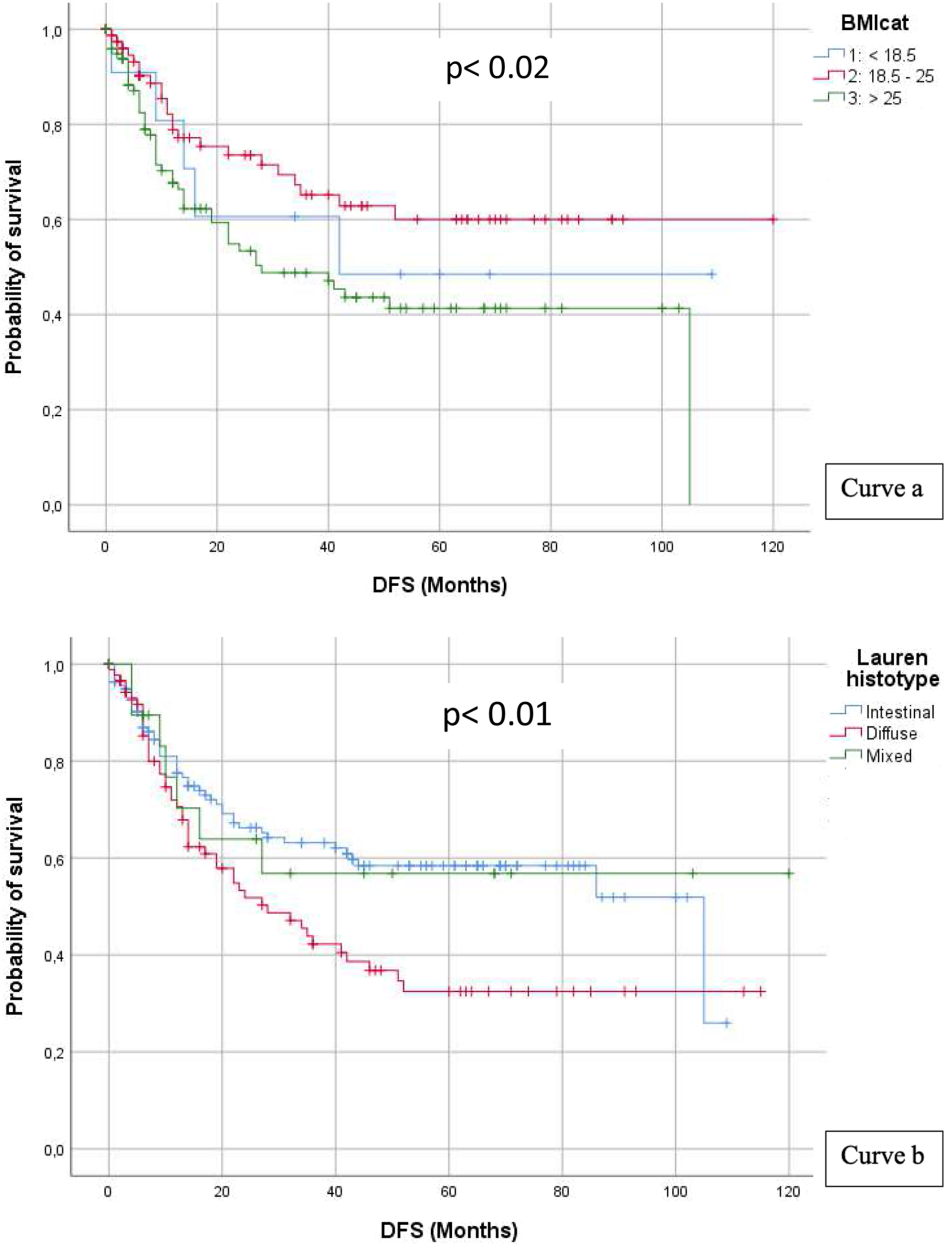
BMI (curve a) and Lauren histotype (curve b).

Preoperative body mass index was separated into three distinct categories: ≤18.5, >18.5–<25, and ≥25, which accounted for 13.6% (*n* = 36), 41.2% (*n* = 108), and 45.2% (*n* = 120) of all patients, respectively. Mean DFS was shorter for under- or overweight patients (61.9 and 52.5 months, respectively) with respect to the normal weight group (79.6 months); the log-rank test showed a statistically significant difference (*p* < 0.02) (see Figure 2 curve a).

#### 3.2.2 Lauren histotype

Tumour histology in the Lauren classification is divided into intestinal and diffuse types; a third category, mixed type, which presents both intestinal and diffuse characteristics, was also analysed. Intestinal type comprised 56.8% of cases (*n* = 150), diffuse type comprised 35.2% of cases (*n* = 93), and mixed type comprised 8.0% of cases (*n* = 21). Mean DFS was shorter for diffuse histotype vs. intestinal (50.2 vs. 67.4 months), whereas mixed type showed an intermediate behaviour; the log-rank test showed a statistically significant difference (*p* < 0.01) (see Figure 2 curve b).

#### 3.2.3 Lymph node positivity and LNR

An average of 27.4 lymph nodes (SD ± 14.9, range 15–86) were retrieved for the final examination, and metastases were found in 223 (84.4%) cases (*p* < 0.001).

The prognostic value of LNR was further investigated in terms of DFS. LNR was divided into four categories according to Marchet et al. (LN1 = 0; LN2 = >0–0.1; LN3 = >0.1–0.25; LN4 = >0.25).

**Figure 3:**
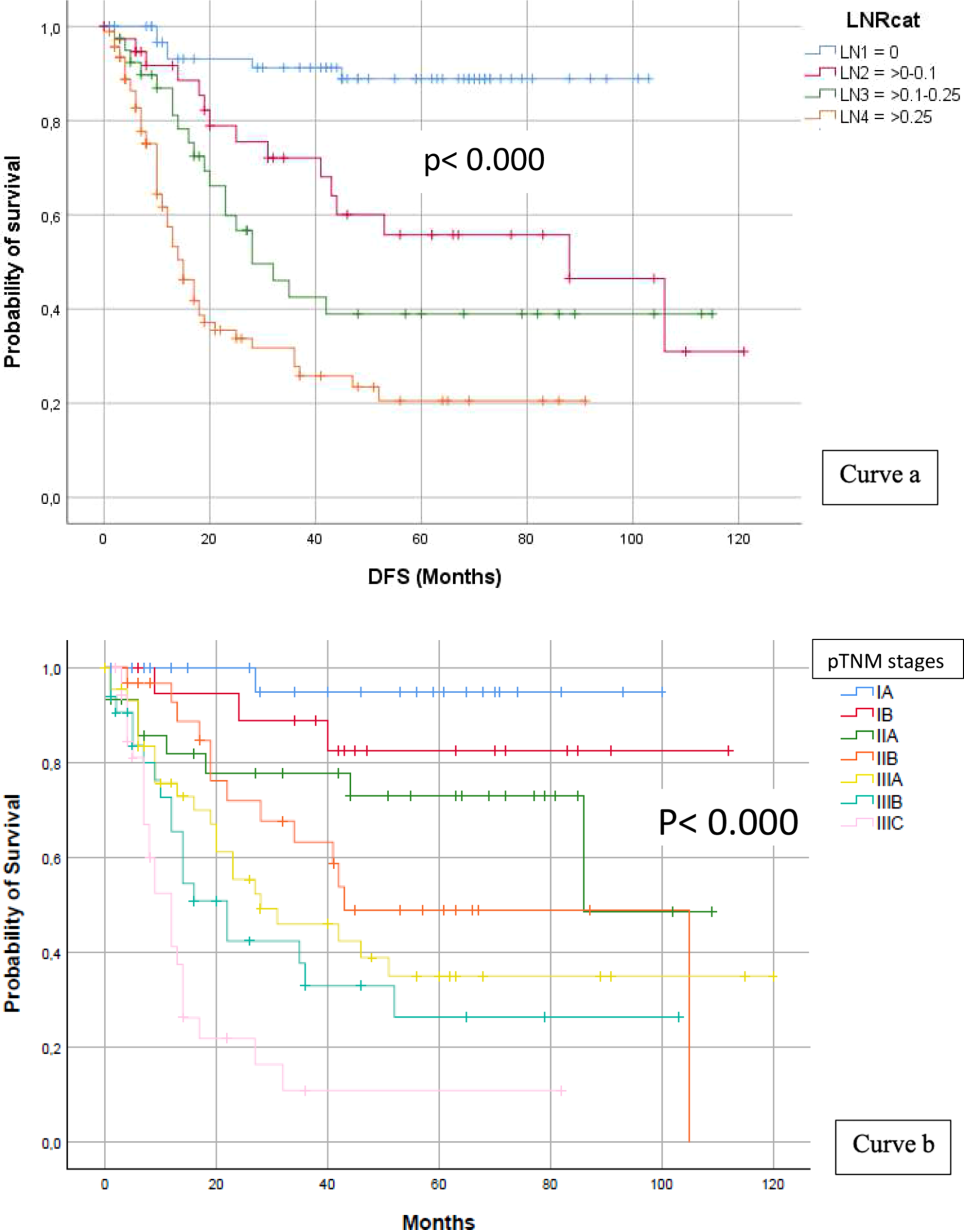
LN ratio and DFS (curve a) and pTNM stages and DFS (curve b).

LN1 accounted for 79 patients (29.9%), LN2 for 42 (16.0%), LN3 for 43 (16.3%), and LN4 for 100 (37.8%). The mean DFS was 93.8 months for LN1, 74.1 months for LN2, 56.9 months for LN3, and 31.0 months for LN4. DFS curves behaved consistently, showing 5-year rates of 88.9%, 55.8%, 39.0%, and 20.5%, respectively; the log-rank test showed a statistically significant difference (*p* < 0.0001) (see Figure 3 curve a).

#### 3.2.4 pTNM

The last TNM classification [UICC classification, 8th ed. (11)] divides patients into eight pathologic stage groups, with the number of patients progressively increasing from stage IA to stage IIIC; consequently, the number of recurrences and lower mean survival time follow a corresponding pattern. DFS survival curves coherently separated from each other, showing 5-year rates of 95.8 (IA), 83.5 (IB), 72.8 (IIA), 48.9 (IIB), 33.9 (IIIA), 26.4 (IIIB), and 10.9 (IIIC), respectively (log-rank test *p* < 0.0001). Most of the recurrence events occurred within 24 months of the intervention; however, remarkably long survival periods were still achievable even in very advanced stages (see Figure 3 curve b).

#### 3.2.5 Histological grading, vascular budding, and perineural and margin infiltration

Grading showed steady progress; the majority of patients developed higher grades and more recurrences; and a similar pattern was observed for vascular budding, whereas most patients did not experience perineural infiltration.

**Figure 4:**
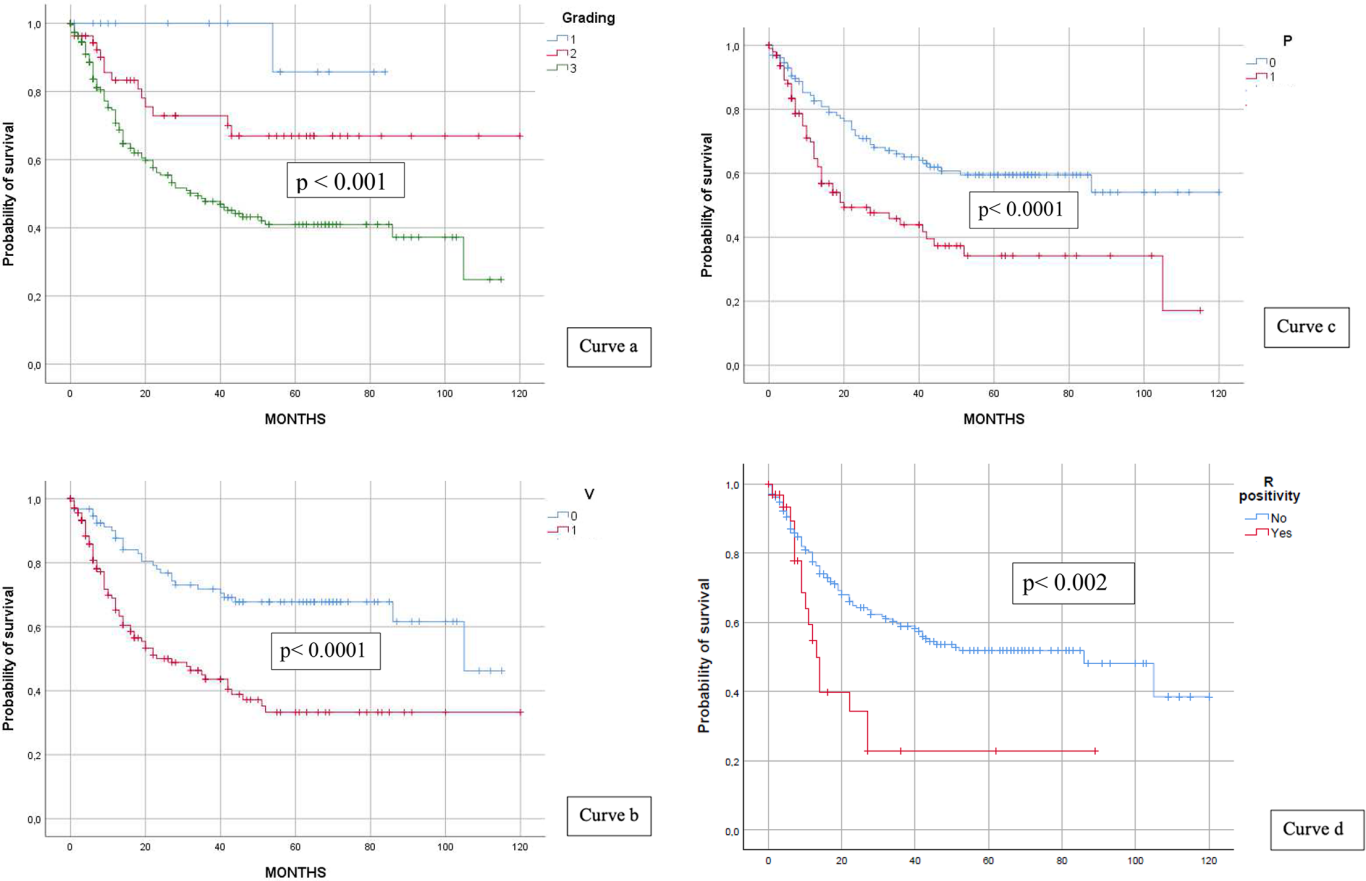
Grading and DFS (curve a), vascular budding and DFS (curve b), perineural infiltration and DFS (curve c), and R positivity and DFS (curve d).

DFS survival curves for each factor were drawn (see curves a, b, c, and d in Figure 4), and differences between groups (i.e., recurrence vs. no recurrence) consistently showed statistical significance (grading *p* < 0.001; vascular budding and perineural infiltration *p* < 0.0001). Margin positivity, although low in percentage (16% vs. 84%), resulted in an expected lower survival (median 83 vs. 13 months, *p* < 0.002). Mean 5-year rates were 85.7% for G1, 66.9% for G2, and 41% for G3, respectively, whereas these rates were 33.3% for V+, 35.2% for P+, and 22% for R+.

#### 3.2.6 Immune inflammation indices NLR, PLR, and LMR

**Figure 5:**
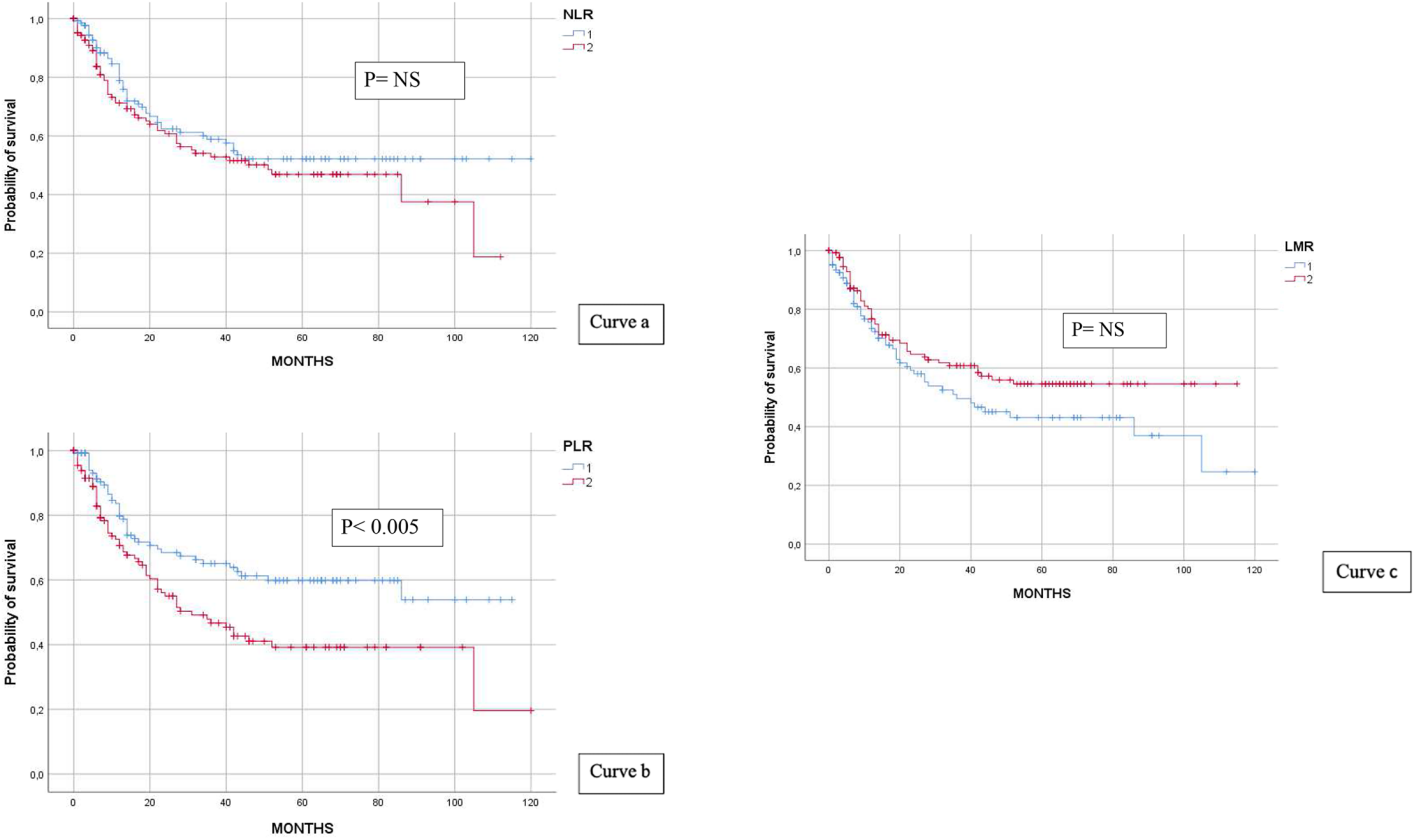
NLR and DFS (curve a), PLR and DFS (curve b), and LMR and DFS (curve c).

Continuous variables of each index were retrieved, and median values (NLR: 2.33; PLR: 134.55; LMR: 4.01) served as a dichotomy for categorising patients (median value ≥ or ≤). Only PLR had an impact on DFS (*p* < 0.005), whereas NLR and LMR had no impact See Figure 5 curves a, b and c.

#### 3.2.7 Complications

Complications occurred in 93 patients (35.2%), of which 36 (39%) were classified as Clavien–Dindo I type, 42 (45.1%) as type II, 6 (6.4%) as type IIIa, 6 (6.4%) as type IIIb, 2 (2.1%) as type IVa, and 1 (1%) as type V. Of all patients with complications, 24 (25.8%) experienced a recurrence of GC, whereas 69 (74.2%) did not show any recurrence. Complications as a group exerted no impact on DFS (log-rank = NS) (see Figure 4 curve a).

#### 3.2.8 Postoperative chemotherapy

**Figure 6:**
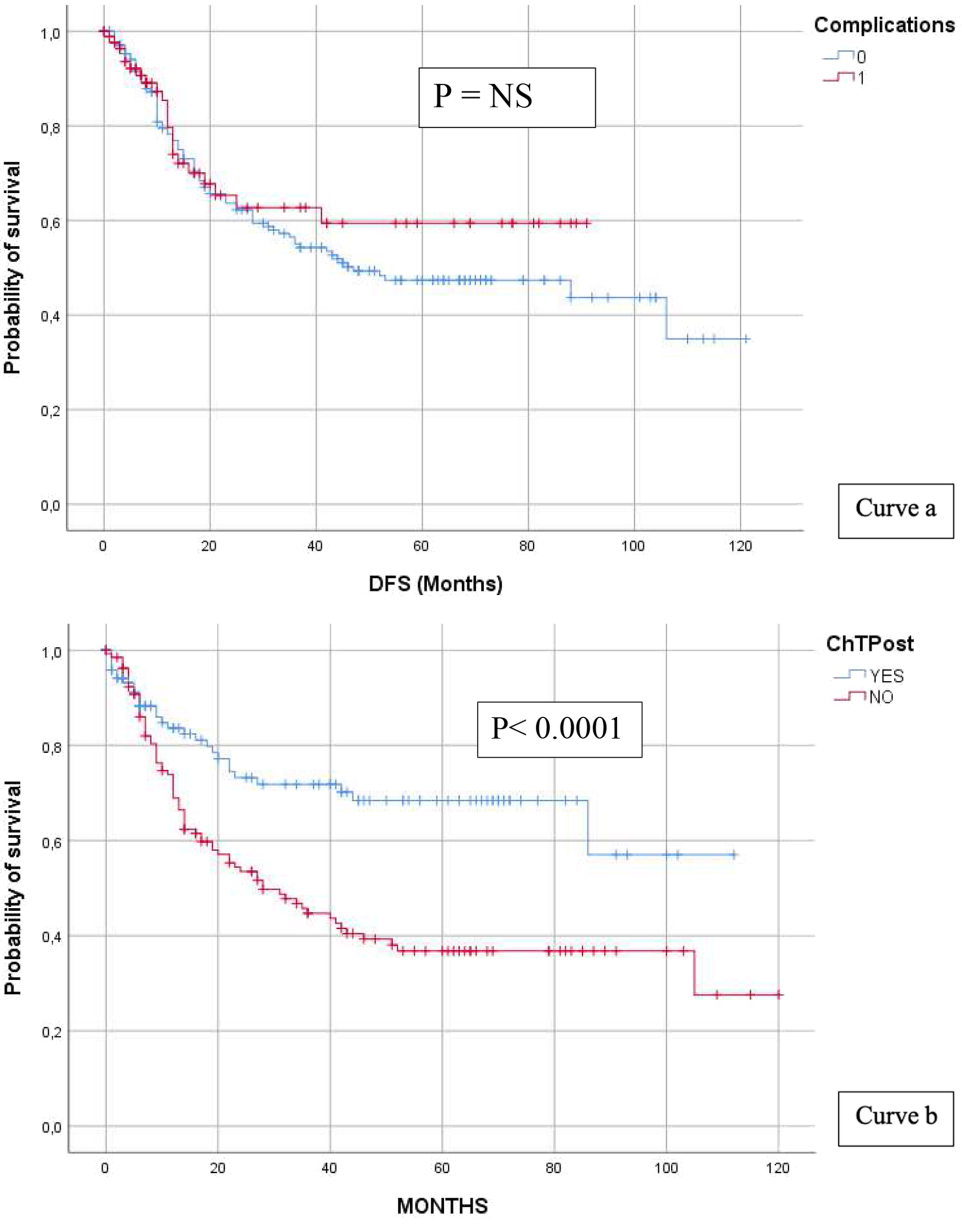
Complications and DFS (curve a) and post-operative chemotherapy and DFS (curve b).

A total of 50.9% of all patients underwent at least a 3-month course of adjuvant chemotherapy. Exclusion criteria were advanced age, ECOG PS ≥ 2, major liver, renal, cardiac, or haematological alterations. The mean DFS with vs. without postoperative chemotherapy was 78.34 vs. 53.96 months, respectively; the 5-year DFS rate was 68.4 vs. 36.8%, respectively, *p* < 0.0001 (see Figure 6 curve b).

### 3.3 Risk factors influencing survival outcomes: univariate and multivariate analyses

Cox univariate and multivariate regression analyses were performed on several examined characteristics to assess their impact as independent risk factors for DFS.

In the univariate analysis, all analysed factors, with the exception of histotype and LMR, showed statistical significance as prognostic factors for DFS. In multivariate analysis, only BMI [*p* < 0.007, hazard ratio (HR) = 1.811], LNR (*p* < 0.002, HR = 1.572), and pTNM (*p* < 0.004, HR = 1.271) showed hazard risk ratios as independent predictors of poor DFS in our series.

Table 2 shows the results of univariate and multivariate Cox regression analyses of risk factors for DFS.

**Table 2:** Univariate and multivariate Cox regression analyses of risk factors for DFS.

Variables	HR	Univariate 95% CI	*P*	HR	Multivariate 95% CI	*p*
Histotype	1.239	0.933–1.645	ns			
BMI	1.495	1.003–2.228	<0.048	1.811	1.173–2.797	<0.007
LNR	1.859	1.412–2.449	<0.001	1.572	1.185–2.086	<0.002
pTNM	1.524	1.369–1.695	<0.001	1.271	1.079–1.498	<0.004
Grading	2.025	1.228–3.340	<0.006			
Vascular invasion	2.596	1.669–4.038	<0.001			
Perineural invasion	2.055	1.368–3.086	<0.001			
R positivity	2.240	1.318–3.807	<0.003			
NLR	1.798	1.154–2.801	<0.01			
PLR	1.975	1.263–3.087	<0.003			
LMR	0.716	0.463–1.109	ns			
Adjuvant chemotherapy	2.197	1.429–3.378	<0.001			

## 4 Discussion

In this retrospective study, we evaluated the DFS of patients with advanced GC who underwent radical resection in a real-life clinical setting at a single cancer centre over a period of 10 years. Advanced GC is an aggressive disease with a high mortality rate. A successful multimodal combination of therapies still relies on radical surgery to achieve long-term survival rates. Findings from our retrospective series, which included 264 patients, confirm the importance of analysing risk factors capable of influencing survival after resection. In particular, preliminary analysis revealed that BMI, LN ratio, stage of disease, grading, vascular budding, perineural and margin infiltration, platelet-to-lymphocyte ratio, and adjuvant chemotherapy all impacted significantly.

Levels of BMI have been associated with lower survival in cancer patients, a trend observed in over- or underweight patients in our series. A meta-analysis of cohort studies also supported that overweight and obesity were associated with an increased risk of GC (12). Conversely, Wong et al. reported a lower DFS in underweight patients in the United States, although BMI did not show significance as a risk factor for DFS in their multivariate analysis (13). In our study, we found that lower BMI was associated with decreased survival for the whole group of GC patients, although this association is rarely reported in the literature. It is still unknown whether the correlation between BMI and GC is casual. Hyperinsulinemia, frequently found among overweight/obese people, represents a risk factor for developing GC. Two Japanese studies showed that impaired fasting blood glucose and high haemoglobin A1c are associated with an increased risk of developing GC in HP-positive individuals (14, 15). Lately, results from studies on sarcopenia and obese sarcopenia have strengthened the assumptions that a strong relationship exists between tissue and body composition and GC (16).

In our study, BMI emerged as a significant prognostic factor, exhibiting an even greater impact on disease-free survival than lymph node ratio, or pTNM, in multivariate analysis.

Different types of Lauren histotypes can predict different prognoses in GC patients, affecting both survival and response to chemotherapy.

In our study, we found a better outcome in terms of DFS for patients affected by intestinal-type GC. The intestinal type, by itself, is typically connected with a better outcome, and Petrelli et al. suggested that it could be used for stratification purposes in future clinical trials (17). Jiménez Fonseca et al. studied the different behaviours between intestinal-type and diffuse-type HER2-negative advanced GC within the AGAMENON National Cancer Registry (patients collected from 30 Spanish and one Chilean centre) (18). They found that intestinal-type tumours were more chemosensitive, in particular when a triple regimen was used with docetaxel regimens. This finding was supported by data published from the FLOT trial (19). Also, Chen et al. published a retrospective study involving 3,071 patients, the majority of whom had intestinal-type rather than diffuse-type or mixed-type GC. Intestinal-type GC led to a better 5-year overall survival rate (57.7%) than diffuse-type (45.6%) or mixed-type (43.4%) GC (20). Recently, histological studies focusing on the significance of PDCs (poorly differentiated clusters) in GC have provided a deeper insight into the strong association between cancer cell types, their behaviour, and prognosis (21).

Vascular invasion and perineural and margin infiltration are usually associated with a poor prognosis. Vascular invasion, in fact, lays the groundwork for the metastatic process; additionally, perineural and margin infiltration are commonly associated with poor prognostic outcomes. Liebig et al. and De Franco et al. both found in a multivariate analysis that PNI is an independent prognostic factor for intestinal-type but not for diffuse-type and mixed-type GC (22, 23), although we found no significance as a risk factor for DFS in our series.

Platelet-to-lymphocyte ratio, as well as neutrophil-to-lymphocyte ratio and monocyte-to-lymphocyte ratio, is a prognostic marker associated with the systemic inflammatory response. A vigorous immune system response of the host has a great impact and is associated with a better outcome, independently from the tumour stage (24). Our data confirmed the association between a high PLR and a lower DFS.

D2 lymph node dissection is of paramount importance for achieving a radical gastrectomy and is nowadays considered the standard of care for GC worldwide. Although the safety and utility of extended lymph node dissection have been long debated in Europe and the United States, D2 dissection is recommended based on several trials, particularly after the Dutch studies (25, 26), and the UICC/TNM staging system coherently incorporates these requests. LNR has been proposed as a surrogate tool for identifying patients with a worse prognosis, and cutoff values divided into four categories, according to Marchet et al. (27, 28), are conveniently used for this purpose. Also, in a recently performed analysis, Woo et al. found that the maximum survival is achieved by performing a lymphadenectomy with a minimum of 29 nodes retrieved (29). In our series, an average of 27 lymph nodes were retrieved, and metastases were found in 84.4% of cases (*p* < 0.001). The subsequent survival analysis accurately reflected the extent of lymph node metastasis in progressive LN ratios, which were consistently associated with worse postoperative DFS and a statistically significant risk in multivariate analysis among other factors like grading, perineural infiltration, or vascular invasion, which did not emerge as significant.

Finally, the pathological stage of the disease is one of the strongest prognostic determinants of DFS in our series. The relevant survival curves showed that an earlier stage was associated with longer survival, and multivariate analysis strengthens this finding. Although a formal subgroup analysis was not performed, it is likely that patients with more advanced stages and/or better LN ratios who received adjuvant therapy did benefit from longer disease-free survival periods. Administration of adjuvant chemotherapy was significant as a risk factor in univariate analysis in our group of patients, although it did not represent an independent association for DFS in multivariate analysis.

In Asian countries, the standard of care consists of surgery followed by adjuvant chemoradiotherapy. In Western countries, the gold standard for resectable tumours is represented by perioperative chemotherapy. FLOT is nowadays the gold standard of care for gastric and oesophageal locally advanced cancer (30, 31). Results published from the MAGIC trial showed that perioperative chemotherapy is the best standard of care for patients affected by resectable, non-early GC (32). One major limitation of our retrospective analysis is that, over the 2009–2019 period, only a limited number of advanced cases were administered perioperative chemotherapy. This likely represents a “pessimistic” view of our oncology team towards neoadjuvant chemotherapy before the advent of FLOT, which undoubtedly represented a major shift in the oncological treatment of patients with more advanced GC.

GC recurrence is a multifactorial event that relies upon numerous elements, the main contributors being the type of tumour, size at diagnosis, depth of invasion, LN metastasis, radical surgery, and Borrmann classification (33, 34). The majority of recurrent cases are usually reported within 2 years from intervention (35), as observed in our series, where BMI, LNR, and pTNM stage were independent risk factors for DFS.

## 5 Conclusion

GC represents one of the most impacting tumours for survival in many countries; however, over the last two decades, newer chemotherapies and advancements in surgical techniques have dramatically changed the perspectives of patients affected by this disease.

With our longitudinal study spanning over the last 10 years of activity in our cancer centre, we have tried to improve the analyses of factors impacting the survival of patients, particularly considering the period from diagnosis to the recurrence of the disease.

One point of strength of our study is that, coherently with other larger published series in different countries and areas, some characteristics like BMI and factors influencing immunity, type, and diffusion of cancer, lymph node status, and chemotherapy were vastly involved in determining disease-specific survival after radical resection.

A limitation is that the time period from 2009 to 2019 predates three major advances, namely, the diffusion of FLOT perioperative chemotherapy, preoperative US endoscopy, and immunotherapy; these advancements have shown a strong effect on the OS and DFS outcomes of GC patients.

Also, it was not possible to show data on comorbidities and 30- and 90-day morbidity, which may have shed light on the intensity of care necessities in an aging population.

Whether or not the patient had received a laparoscopic rather than an open procedure was not a matter of analysis, as the number of patients was not consistent enough to draw any conclusions different from what the literature has already demonstrated (36). A radical and oncologically correct gastrectomy and lymphadenectomy remain the most important factors for survival.

## Data Availability

The raw data supporting the conclusions of this article will be made available by the authors, without undue reservation.

## References

[1] Rawla P Barsouk A. Epidemiology of gastric cancer: global trends, risk factors and prevention. Prz Gastroenterol. (2019) 14(2):89–103. 10.5114/pg.2018.80001 30944675 PMC6444111

[2] Machlowska J Baj J Sitarz M Maciejewski R Sitarz R. Gastric cancer: epidemiology, risk factors, classification, genomic characteristics and treatment strategies. Int J Mol Sci. (2020) 21(11):4012. 10.3390/ijms21114012 32512697 PMC7312039

[3] Petrovchich I Ford JM. Genetic predisposition to gastric cancer. Semin Oncol. (2016) 43(5):554–9. 10.1053/j.seminoncol.2016.08.006 27899187

[4] Marrelli D Pedrazzani C Morgagni P de Manzoni G Pacelli F Coniglio A Changing clinical and pathological features of gastric cancer over time. Br J Surg. (2011) 98(9):1273–83. 10.1002/bjs.7528 21560122

[5] Japanese Gastric Cancer Association. Japanese Gastric cancer treatment guidelines 2018 (5th edition). Gastric Cancer. (2021) 24(1):1–21. 10.1007/s10120-020-01042-y 32060757 PMC7790804

[6] Waddell T Verheij M Allum W Cunningham D Cervantes A Arnold D. Gastric cancer: ESMO-ESSO-ESTRO clinical practice guidelines for diagnosis, treatment and follow-up. Eur J Surg Oncol. (2014) 40(5):584–91. 10.1016/j.ejso.2013.09.020 24685156

[7] Bauer K Manzini G Henne-Bruns D Buechler P. Perioperative chemotherapy for advanced gastric cancer-results from a tertiary-care hospital in Germany. World J Gastrointest Oncol. (2020) 12(5):559–68. 10.4251/WJGO.V12.I5.559 32461787 PMC7235186

[8] Dindo D Demartines N Clavien PA. Classification of surgical complications. A new proposal with evaluation in a cohort of 6336 patients and results of a survey. Ann Surg. (2004) 240(2):205–13. 10.1097/01.sla.0000133083.54934.ae 15273542 PMC1360123

[9] De Manzoni G Marrelli D Baiocchi GL Morgagni P Saragoni L Degiuli M The Italian Research Group for Gastric Cancer (GIRCG) guidelines for gastric cancer staging and treatment: 2015. Gastric Cancer. (2017) 20(1):20–30. 10.1007/s10120-016-0615-3 27255288

[10] Escrig Sos J Gómez Quiles L Maiocchi K. The 8th edition of the AJCC-TNM classification: new contributions to the staging of esophagogastric junction cancer. Cir Esp. (2019) 97(8):432–7. 10.1016/j.ciresp.2019.03.006 31029372

[11] Liu JY Peng CW Yang XJ Huang CQ Li Y. The prognosis role of AJCC/UICC 8th edition staging system in gastric cancer, a retrospective analysis. Am J Transl Res. (2018) 10(1):292–303. 29423014 PMC5801367

[12] Li F Du H Li S Liu J. The association between metabolic syndrome and gastric cancer in Chinese. Front Oncol. (2018) 8:326. 10.3389/fonc.2018.00326 30191141 PMC6116659

[13] Wong J Rahman S Saeed N Lin H Almhanna K Shridhar R Effect of body mass index in patients undergoing resection for gastric cancer: a single center US experience. J Gastrointest Surg. (2014) 18(3):505–11. 10.1007/s11605-014-2455-y 24443204

[14] Yamagata H Kiyohara Y Nakamura S Kubo M Tanizaki Y Matsumoto K Impact of fasting plasma glucose levels on gastric cancer incidence in a general Japanese population: the Hisayama study. Diabetes Care. (2005) 28(4):789–94. 10.2337/diacare.28.4.789 15793174

[15] Ikeda F Doi Y Yonemoto K Ninomiya T Kubo M Shikata K Hyperglycemia increases risk of gastric cancer posed by Helicobacter pylori infection: a population-based cohort study. Gastroenterology. (2009) 136(4):1234–41. 10.1053/j.gastro.2008.12.045 19236964

[16] Ricciardolo AA De Ruvo N Serra F Prampolini F Solaini L Battisti S Strong impact of sarcopenia as a risk factor of survival in resected gastric cancer patients: first Italian report of a Bicentric study. Updates Surg. (2022) 74(1):283–93. 10.1007/s13304-021-01175-4 34699033

[17] Petrelli F Berenato R Turati L Mennitto A Steccanella F Caporale M Prognostic value of diffuse versus intestinal histotype in patients with gastric cancer: a systematic review and meta-analysis. J Gastrointest Oncol. (2017) 8(1):148–63. 10.21037/jgo.2017.01.10 28280619 PMC5334055

[18] Jiménez Fonseca P Carmona-Bayonas A Hernández R Custodio A Cano JM Lacalle A Lauren subtypes of advanced gastric cancer influence survival and response to chemotherapy: real-world data from the AGAMENON national cancer registry. Br J Cancer. (2017) 117(6):775–82. 10.1038/bjc.2017.245 28765618 PMC5589993

[19] Al-Batran SE Homann N Pauligk C Goetze TO Meiler J Kasper S Perioperative chemotherapy with fluorouracil plus leucovorin, oxaliplatin, and docetaxel versus fluorouracil or capecitabine plus cisplatin and epirubicin for locally advanced, resectable gastric or gastro-oesophageal junction adenocarcinoma (FLOT4): a randomized, phase 2/3 trial. Lancet. (2019) 393(10184):1948–57. 10.1016/S0140-6736(18)32557-1 30982686

[20] Chen YC Fang WL Wang RF Liu CA Yang MH Lo SS Clinicopathological variation of lauren classification in gastric cancer. Pathol Oncol Res. (2016) 22(1):197–202. 10.1007/s12253-015-9996-6 26502923

[21] Sorrentino L De Ruvo N Serra F Salati M Ricciardolo AA Bonetti LR Role of poorly differentiated cluster in gastric cancer: is it a new prognosis factor? Scand J Gastroenterol. (2022) 57(1):44–9. 10.1080/00365521.2021.1974932 34524049

[22] Liebig C Ayala G Wilks JA Berger DH Albo D. Perineural invasion in cancer: a review of the literature. Cancer. (2009) 115(15):3379–91. 10.1002/cncr.24396 19484787

[23] De Franco L Marrelli D Voglino C Vindigni C Ferrara F Di Mare G Prognostic value of perineural invasion in resected gastric cancer patients according to lauren histotype. Pathol Oncol Res. (2018) 24(2):393–400. 10.1007/s12253-017-0257-8 28555306

[24] Roxburgh CSD McMillan DC. Cancer and systemic inflammation: treat the tumour and treat the host. Br J Cancer. (2014) 110(6):1409–12. 10.1038/bjc.2014.90 24548867 PMC3960633

[25] Bonenkamp JJ Hermans J Sasako M Welvaart K Songun I Meyer S Extended lymph-node dissection for gastric cancer. N Engl J Med. (1999) 340(12):908–14. 10.1056/nejm199903253401202 10089184

[26] Hartgritik HH Van De Velde CJH Putter H Bonenkamp JJ Klein Kanenbarg E Songun I Extended lymph node dissection for gastric cancer: who may benefit? Final results of the randomized Dutch gastric cancer group trial. J Clin Oncol. (2004) 22(11):2069–77. 10.1200/JCO.2004.08.026 15082726

[27] Marchet A Mocellin S Ambrosi A Morgagni P Garcea D Marrelli D The ratio between metastatic and examined lymph nodes (N ratio) is an independent prognostic factor in gastric cancer regardless of the type of lymphadenectomy: results from an Italian multicentric study in 1853 patients. Ann Surg. (2007) 245(4):543–52. 10.1097/01.sla.0000250423.43436.e1 17414602 PMC1877031

[28] Marchet A Mocellin S Ambrosi A Morgagni P Garcea D Marrelli D The prognostic value of N-ratio in patients with gastric cancer: validation in a large, multicenter series. Eur J Surg Oncol. (2007) 245(4):543–52. 10.1016/j.ejso.2007.04.018 17566691

[29] Woo Y Goldner B Ituarte P Lee B Melstrom L Son T Lymphadenectomy with Optimum of 29 lymph nodes retrieved associated with improved survival in advanced gastric cancer: a 25,000-patient international database study. J Am Coll Surg. (2017) 224(4):546–55. 10.1016/j.jamcollsurg.2016.12.015 28017807 PMC5606192

[30] Al-Batran SE Hofheinz RD Pauligk C Kopp HG Haag GM Luley KB Histopathological regression after neoadjuvant docetaxel, oxaliplatin, fluorouracil, and leucovorin versus epirubicin, cisplatin, and fluorouracil or capecitabine in patients with resectable gastric or gastro-oesophageal junction adenocarcinoma (FLOT4-AIO). Lancet Oncol. (2016) 17(12):1697–708. 10.1016/S1470-2045(16)30531-9 27776843

[31] Ilson DH. Advances in the treatment of gastric cancer: 2019. Curr Opin Gastroenterol. (2019) 35(6):551–4. 10.1097/MOG.0000000000000577 31436556

[32] Cunningham D Allum WH Stenning SP Thompson JN Van de Velde CG Nicolson M Perioperative chemotherapy versus surgery alone for resectable gastroesophageal cancer. N Engl J Med. (2006) 355(1):11–20. 10.1056/nejmoa055531 16822992

[33] Song XH Zhang WH Kai-Liu CX Chen XL Zhao LY Chen XZ Prognostic impact of Borrmann classification on advanced gastric cancer: a retrospective cohort from a single institution in western China. World J Surg Oncol. (2020) 18(1):204. 10.1186/s12957-020-01987-5 32792016 PMC7427284

[34] Wang SY Yeh CN Lee HL Liu YY Chao TC Hwang TL Clinical impact of positive surgical margin status on gastric cancer patients undergoing gastrectomy. Ann Surg Oncol. (2009) 16(10):2738–43. 10.1245/s10434-009-0616-0 19636636

[35] D’Angelica M Gonen M Brennan MF Turnbull AD Bains M Karpeh MS. Patterns of initial recurrence in completely resected gastric adenocarcinoma. Ann Surg. (2004) 240(5):808–16. 10.1097/01.sla.0000143245.28656.15 15492562 PMC1356486

[36] Kim HH Han SU Kim MC Wyung WJ Kim W Lee HJ Long-term results of laparoscopic gastrectomy for gastric cancer: a large-scale case-control and case-matched Korean multicenter study. J Clin Oncol. (2014) 32(7):627–33. 10.1200/JCO.2013.48.8551 24470012

